# Optimising Treatment Outcomes for Children and Adults Through Rapid Genome Sequencing of Sepsis Pathogens. A Study Protocol for a Prospective, Multi-Centre Trial (DIRECT)

**DOI:** 10.3389/fcimb.2021.667680

**Published:** 2021-06-23

**Authors:** Adam D. Irwin, Lachlan J. M. Coin, Patrick N. A. Harris, Menino Osbert Cotta, Michelle J. Bauer, Cameron Buckley, Ross Balch, Peter Kruger, Jason Meyer, Kiran Shekar, Kara Brady, Cheryl Fourie, Natalie Sharp, Luminita Vlad, David Whiley, Scott A. Beatson, Brian M. Forde, David Paterson, Julia Clark, Krispin Hajkowicz, Sainath Raman, Seweryn Bialasiewicz, Jeffrey Lipman, Luregn J. Schlapbach, Jason A. Roberts

**Affiliations:** ^1^ UQ Centre for Clinical Research, The University of Queensland, Brisbane, QLD, Australia; ^2^ Infection Management and Prevention Service, Queensland Children’s Hospital, Brisbane, QLD, Australia; ^3^ Department of Microbiology and Immunology, University of Melbourne at The Peter Doherty Institute for Infection and Immunity, Melbourne, VIC, Australia; ^4^ Institute for Molecular Bioscience, The University of Queensland, Brisbane, QLD, Australia; ^5^ Intensive Care Unit, Princess Alexandra Hospital, Brisbane, QLD, Australia; ^6^ Adult Intensive Care Services and Critical Care Research Group, The Prince Charles Hospital, Brisbane, QLD, Australia; ^7^ Department of Infectious Diseases, Royal Brisbane and Women's Hospital, Brisbane, Brisbane, QLD, Australia; ^8^ Paediatric Intensive Care Unit, Queensland Children’s Hospital, Children’s Health Queensland, Brisbane, QLD, Australia; ^9^ School of Chemistry and Molecular Biosciences, The University of Queensland, Brisbane, QLD, Australia; ^10^ Department of Pediatric and Neonatal Intensive Care, University Children’s Hospital Zurich, Zurich, Switzerland

**Keywords:** antimicrobials, nanopore sequencing, antimicrobial resistance, sepsis diagnostics, personalised dosing, trial protocol

## Abstract

**Background:**

Sepsis contributes significantly to morbidity and mortality globally. In Australia, 20,000 develop sepsis every year, resulting in 5,000 deaths, and more than AUD$846 million in expenditure. Prompt, appropriate antibiotic therapy is effective in improving outcomes in sepsis. Conventional culture-based methods to identify appropriate therapy have limited yield and take days to complete. Recently, nanopore technology has enabled rapid sequencing with real-time analysis of pathogen DNA. We set out to demonstrate the feasibility and diagnostic accuracy of pathogen sequencing direct from clinical samples, and estimate the impact of this approach on time to effective therapy when integrated with personalised software-guided antimicrobial dosing in children and adults on ICU with sepsis.

**Methods:**

The DIRECT study is a pilot prospective, non-randomized multicentre trial of an integrated diagnostic and therapeutic algorithm combining rapid direct pathogen sequencing and software-guided, personalised antibiotic dosing in children and adults with sepsis on ICU.

**Participants and interventions:**

DIRECT will collect microbiological and pharmacokinetic samples from approximately 200 children and adults with sepsis admitted to one of four ICUs in Brisbane. In Phase 1, we will evaluate Oxford Nanopore Technologies MinION sequencing direct from blood in 50 blood culture-proven sepsis patients recruited from consecutive patients with suspected sepsis. In Phase 2, a further 50 consecutive patients with suspected sepsis will be recruited in whom MinION sequencing will be combined with Bayesian software-guided (ID-ODS) personalised antimicrobial dosing.

**Outcome measures:**

The primary outcome is time to effective antimicrobial therapy, defined as trough drug concentrations above the MIC of the pathogen. Secondary outcomes are diagnostic accuracy of MinION sequencing from whole blood, time to pathogen identification and susceptibility testing using sequencing direct from whole blood and from positive blood culture broth.

**Discussion:**

Rapid pathogen sequencing coupled with antimicrobial dosing software has great potential to overcome the limitations of conventional diagnostics which often result in prolonged inappropriate antimicrobial therapy. Reduced time to optimal antimicrobial therapy may reduce sepsis mortality and ICU length of stay. This pilot study will yield key feasibility data to inform further, urgently needed sepsis studies. Phase 2 of the trial protocol is registered with the ANZCTR (ACTRN12620001122943).

**Trial registration:**

Registered with the Australia New Zealand Clinical Trials Registry Number ACTRN12620001122943

## Introduction

Sepsis contributes significantly to morbidity and mortality of children and adults worldwide with an incidence that is increasing in line with global estimates (Schlapbach et al., 2015; Fleischmann-Struzek et al., 2018; Rudd et al., 2020). Close to 20,000 Australians develop sepsis every year, in whom approximately 5,000 deaths occur. The cost to the Australian healthcare system has been estimated to exceed AUD$846 million annually, with ICU treatment required for ongoing organ dysfunction resulting in the majority of costs (https://www.georgeinstitute.org/sites/default/files/documents/stopping-sepsis-national-action-plan.pdf). Appropriate antibiotic therapy is demonstrably effective in improving outcomes in sepsis (Kumar et al., 2009; Seymour et al., 2017; Levy et al., 2018; Evans et al., 2018). However, the appropriateness of antibiotic therapy in sepsis is still predominantly determined by traditional susceptibility testing of cultured organisms. These conventional techniques have a yield below 50% in most ICU studies and take days to complete (Agyeman et al., 2017). It is often 48 to 72 hours before antibiotic susceptibility of pathogens are known, leading to substantial delays in optimizing antibiotic therapy, and to overuse of broad spectrum antibiotics. This timeframe for the identification of a causative organism and its susceptibility profile may result in a prolonged period of inappropriate empirical treatment. In an era of increasing antibiotic resistance, such suboptimal therapy can contribute to increased resistance, and worse patient-centred outcomes. In adults, it has been observed that Gram-negative healthcare-associated infections are associated with significant delays in effective antibiotic therapy. Such delays prolong the requirement for intensive care and lead to increased morbidity, mortality and cost (Moehring et al., 2013). A day in ICU costs approximatively AUD$6,000 for ICU infrastructure alone. Hence strategies with the potential to hasten patient recovery and shorten ICU length of stay have great promise to be cost-effective.

A rapid, sensitive technique capable of identifying the pathogen responsible for sepsis, along with its susceptibility profile has the potential to substantially improve clinical outcomes through earlier selection of effective antimicrobial therapy. Culture-independent methods which are not limited to specific pathogen targets are appealing. Sequencing-based approaches meet these requirements. In particular, the Oxford Nanopore Technologies (ONT) MinION device provides informative sequence data in real-time (Clarke et al., 2009), offering the potential for rapid pathogen identification along with susceptibility profiles. This effectiveness can be maximised by optimising antibiotic concentrations through individualized dosing (Roberts et al., 2014; Abdul-Aziz et al., 2020).

We propose an integrated approach to personalize antimicrobial therapy in children and adults with sepsis. We will evaluate the impact of portable nanopore sequencing using the ONT MinION device integrated with Bayesian dosing software (ID-ODS) to ensure achievement of maximally effective personalised antibiotic exposures in critically ill children and adults with sepsis.

## Trial Design

The DIRECT study is a pilot prospective, non-randomised multicentre trial of an integrated diagnostic and therapeutic algorithm combining rapid direct pathogen sequencing and software-guided, personalised antibiotic dosing in children and adults with sepsis on ICU. It includes a diagnostic accuracy study of real-time pathogen sequencing direct from patient blood using the ONT MinION device, which will be compared to conventional pathogen sequencing of cultured isolates, and conventional blood culture and phenotypic susceptibility testing. This study protocol follows the Standard Protocol Items: Recommendations for Interventional Trials (SPIRIT) guidelines (Chan et al., 2013). See **Supplementary File 1**.

## Hypothesis

Real-time pathogen sequencing combined with dosing software to identify optimised personalised antimicrobial therapy will reduce the time to effective antimicrobial concentrations in critically ill patients with sepsis leading to improved patient outcomes.

## Aim

To demonstrate the feasibility and diagnostic accuracy of real-time pathogen sequencing direct from clinical samples and estimate the impact of this diagnostic approach on time to effective therapy when integrated with personalised antimicrobial dosing in children and adults on ICU with sepsis.

## Objectives

### Primary Objective

To estimate the reduction in time to effective antibiotic therapy in children and adults with sepsis using rapid pathogen sequencing combined with dosing software when compared to conventional diagnostic and therapeutic regimens.

### Secondary Objectives

i. A demonstration of the feasibility of real-time pathogen sequencing in whole blood samples from critically ill children and adults with sepsis.
ii. An evaluation of the diagnostic accuracy of real-time pathogen sequencing from positive blood cultures taken from critically ill children and adults with sepsis.
iii. An evaluation of the time to pathogen identification comparing direct sequencing versus conventional blood cultures taken from critically ill children and adults with sepsis.
iv. An improvement in the achievement of therapeutic antibiotic concentrations in patients using dosing software versus traditional dosing regimens.

## Methods

### Study Setting

The study will be undertaken in the following participating ICUs in Brisbane, Australia: Royal Brisbane and Women’s Hospital, The Prince Charles Hospital, Princess Alexandra Hospital, Queensland Children’s Hospital.

### Eligibility Criteria

Patients with suspected sepsis or septic shock admitted to participating ICUs with an onset of less than 24hours. A summary of all inclusion and exclusion criteria for participants are provided in **Table 1**.

**Table 1:** Inclusion and exclusion criteria for the DIRECT study.

**Inclusion criteria** Age >1month Admitted to paediatric or adult ICU at one of the participating centres Decision to treat for suspected sepsis, defined as suspected or proven infection with or without confirmed organ dysfunction. Commenced within 24h on intravenous broad-spectrum antibiotics, or within 24h of a change to new antibiotics consistent with treatment for a new episode of suspected sepsis. Blood cultures are being obtained or were obtained within the past 12 hours
**Exclusion criteria:** Inability to gain informed consent during the study period Neonates Death is likely imminent Palliative care patient Renal replacement therapy Extra-corporeal membrane oxygenation

### Interventions

Diagnostic accuracy of MinION nanopore pathogen sequencing will be compared with conventional blood cultures and antimicrobial susceptibility testing. Acknowledging the limited yield of blood cultures in sepsis, a composite reference standard for sepsis will be used. This composite reference standard will incorporate blood culture results, other significant microbiological samples taken at the discretion of the clinical team and clinical and epidemiological features. These composite features will be interpreted independently by two experts in microbiology and infectious disease. In the event of discordance a third expert opinion will adjudicate. Complete reference genomes for matching pathogen isolates will be analysed to evaluate the differences between nanopore direct sequencing and cultured pathogens sequenced conventionally.

### Outcomes

#### Primary Outcome

The primary outcome is time to effective antimicrobial therapy defined as trough drug concentrations above the MIC of the pathogen.

#### Secondary Outcomes

Secondary outcomes are: 1) Diagnostic accuracy of MinION nanopore pathogen sequencing direct from whole blood

2) Time to pathogen identification and susceptibility testing using MinION nanopore pathogen sequencing 

a) direct from whole blood 

b) from positive blood culture broth. 

3) Evaluation of direct MinION nanopore pathogen sequencing predictive capabilities using complete reference genomes from positive blood culture broth.

#### Sample Size and Recruitment

**Figure 1:**
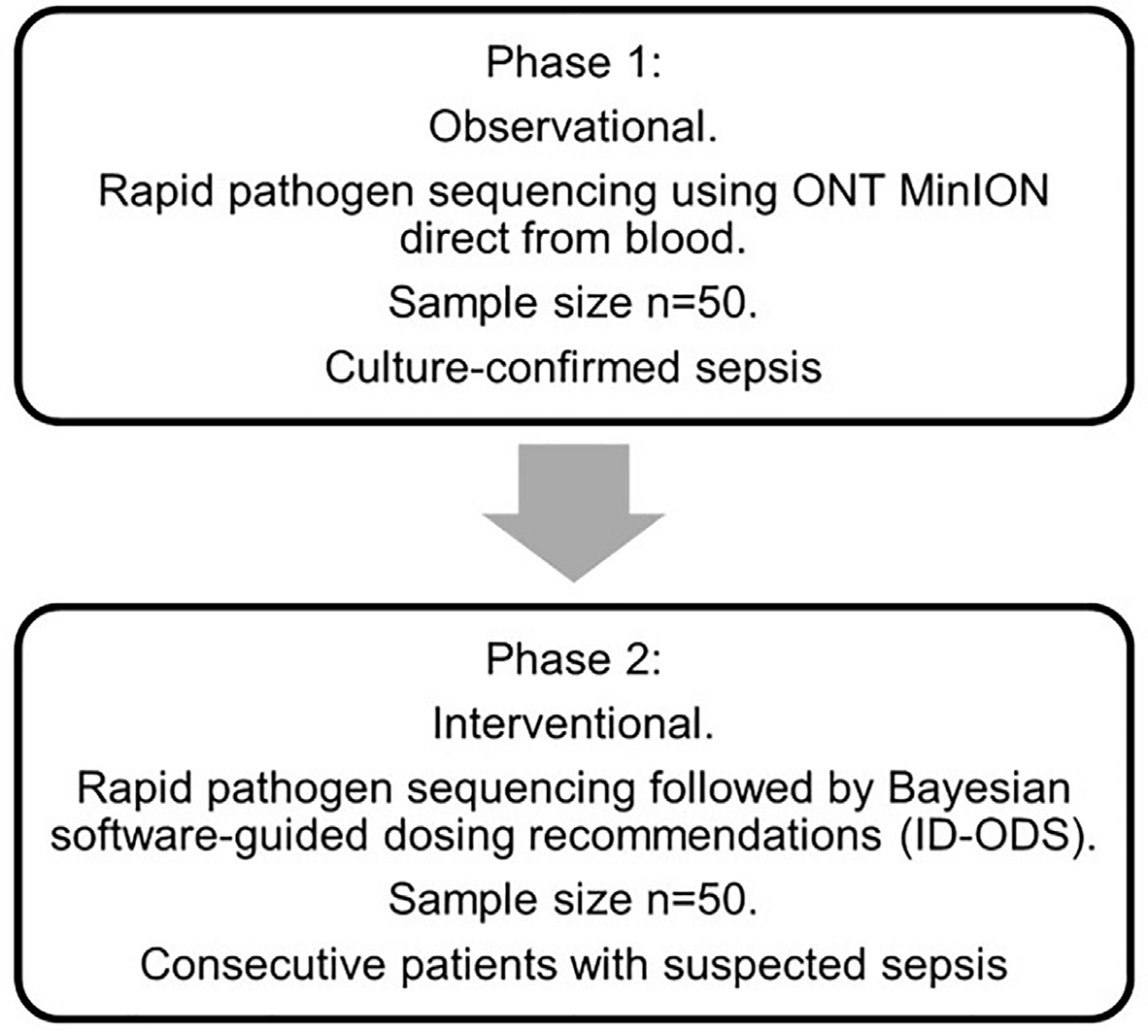
Study flow diagram. Phase 1: In an initial observational phase of the study, participants will be recruited to a diagnostic accuracy study of MinION nanopore pathogen sequencing. The sample size for this phase of the study is 50 patients with blood culture-confirmed sepsis admitted to ICU. Phase 2: In Phase 2, consecutive patients with suspected sepsis admitted to ICU will undergo MinION nanopore pathogen sequencing integrated with personalised antibiotic therapy using a combination of Bayesian dosing software (ID-ODS™) and measured antibiotic plasma concentrations. A senior ICU pharmacist/clinician at each site will lead this software-guided intervention of antimicrobial dose optimisation. All dosing regimens will be checked by both the senior ICU pharmacist and attending ICU consultant prior to prescription. The final decision regarding the use of the optimised dosing of antibiotics will remain at the discretion of the attending ICU consultant. Software-guided dosing will continue until either: 1) the study antibiotic(s) have been ceased by the treating clinician, 2) the patient is discharged from ICU, 3) after 5 days of study antibiotic therapy. If antibiotic therapy is still required thereafter, dosing will be guided by the treating clinician. Adherence to dosing strategies informed by the dosing software will be supported by the use of senior ICU pharmacists trained in the use of this software-guided approach to antimicrobial dose optimisation. All dosing regimens will be checked by both the senior ICU pharmacist and attending ICU consultant prior to prescription, to ensure appropriateness and safety.

The study will be undertaken in two phases (**Figure 1**). Phase 1 will seek to demonstrate the validity of the sequencing method applied to patients with blood culture-confirmed sepsis (n=50). Recruitment of 50 blood culture confirmed-sepsis patients in this phase is anticipated to require the recruitment of approximately 150 patients with suspected sepsis. Phase 2 will then apply these methods to consecutive patients (n=50) admitted to intensive care with suspected sepsis. No randomization of patients will occur. Recruitment to the study commenced 17th March 2020 and will be completed by 30^th^ September 2021. Education sessions were conducted with research groups from each ICU in advance of the study onset, including individual sessions with local research coordinators. Study sample packs, and flow diagrams to aid recruitment were created for participating ICUs. Research coordinators are responsible for screening potentially eligible patients with suspected sepsis on a daily basis. Finally, ethical approval was sought to recruit patients with suspected sepsis on a “consent to continue” basis, reflecting the time-critical nature of sepsis and the importance of obtaining timely research samples.

### Consent

Patients and/or carers will be approached by clinical research staff at each participating ICU and provided with written information regarding the study. A consent to continue process will be used where prospective recruitment would lead to delays in blood sampling and preclude direct comparison with blood cultures. Carers and competent patients will be provided with the opportunity to revoke their consent and have the research blood samples and data securely disposed of prior to analysis. Eligible patients and their carers will be approached to contribute residual blood samples for the evaluation of novel, rapid sepsis diagnostics (commercial and research, culture-dependent and culture-independent).

### Ethics Approval and Consent to Participate

Ethics approval has been granted by the Children’s Health Queensland Hospital and Health Service Human Research Ethics Committee (HREC) [HREC/19/QCHQ/55177]. Written informed consent will be obtained from all participants (or their parent or legal guardian). Approval was granted by the Queensland Civil and Administrative Tribunal [CRL024-19] to include patients unable to consent for themselves under the Guardianship and Administration Act 2000.

### Data Collection and Management

Clinical data on patient demographics, comorbidities, type and focus of infection, severity, clinical progress through ICU and clinical outcomes (including ICU and hospital length of stay and in-hospital mortality) will be captured by clinical research nurses into an online REDCap database hosted by The University of Queensland. Organ dysfunction in children and adults is defined in **Supplementary File 2**. In the event that a paper Case report form is used, forms will be stored in an appropriate locked storage facility with restricted access. A study ID number will be used to link de-identified clinical data with the laboratory samples. Data and samples will be retained in line with the Queensland Health Retention and Disposal Schedule and with the Australian Code for the Responsible Conduct of Research Section 2.1.1. Deidentified genome sequence data will be shared by deposition in a public nucleotide repository (GenBank and Sequence Read Archive). Adherence to the study protocol will be monitored by the Coordinating Principal Investigator, and the steering committee. Patient eligibility, consents, and sample processing will be audited every 6 months at each site. An annual progress report will be submitted to the approving human research ethics committee (HREC) and local research governance officers (RGOs). No adverse events are anticipated, and a Data Safety and Monitoring Board has not been convened.

### Study Samples

Initial study samples will be obtained during the course of the routine clinical sepsis workup. Samples will be stored until consent to participate has been sought, and will then be analysed if consent is given, or destroyed if consent is not given or revoked.

The following study samples will be obtained: i. A single study sample will be taken into an EDTA container at the time of recruitment to the study, and as close to the time of initial blood culture sampling on suspicion of sepsis. (Time=zero). Routine clinical sampling should include appropriate volumes of blood and other clinical samples for culture and susceptibility testing (**Table 2**).

**Table 2:** Appropriate study sample volumes according to age, and sample type. ‘T’ is time of sampling in hours since recruitment.

Age category	EDTA (T=0)	Plasma (T=24,48,72,96h)
Infants and Young children (<5y)	1-2ml	0.5ml
Older children (5-12y)	2-6ml	1-2ml
Adolescents and adults (>12y)	6-10ml	3-5ml

The volume of blood obtained (for both children and adults) will be documented at the time of recruitment and sampling. Consent will be sought to use residual EDTA samples for the evaluation of emerging, rapid methods of bacterial detection and antimicrobial resistance identification.

ii. Plasma samples will be taken at the following time points of antibiotic therapy: at 24 hours, 48 hours, 72 hours and 96 hours post-commencement of antibiotic treatment to determine if target antibiotic plasma concentrations have been reached.

Clinical samples will be processed in line with existing laboratory requirements. Study EDTA samples will be stored at 4°C before shipping to The University of Queensland Centre for Clinical Research (UQCCR) and the Queensland Paediatric Infectious Disease (QPID) laboratory. Bacterial isolates will be stored at -80°C before shipping to UQCCR. Samples will be de-identified before shipping, and labelled with a study ID. Samples at UQCCR will be stored at -80°C until analysis.

### Reference Standard

The diagnostic accuracy of real-time pathogen sequencing using the MinION sequencer for the diagnosis of sepsis will be evaluated against a reference standard of conventional microbiological evaluation using blood culture and susceptibility testing.

### Laboratory Methods

Routine clinical samples will be processed in NATA accredited laboratories at Pathology Queensland. Time to blood culture growth, time to pathogen identification, and time to susceptibility testing completion will be collected.

### Broth Microdilution

Susceptibility testing will be undertaken at The University of Queensland Centre for Clinical Research (UQCCR) using a broth microdilution method (BMD). In house BMD plates will be made using the Hamilton Star liquid handling robot as per European Committee on Antimicrobial Susceptibility Testing (EUCAST) standards and testing will be performed on demand as isolates are made available. Antimicrobials tested by BMD will be chosen by the study investigators and will reflect available and readily used antimicrobials in clinical practice.

### Whole Genome Sequencing of Sepsis Pathogens

Bacterial cells will first be enriched using a differential lysis procedure. Residual human DNA will be degraded using a DNAse treatment, followed by nucleic acid extraction using a rapid commercial-kit based protocol. Whole genome sequencing (WGS) of sepsis pathogen isolates with ONT MinION or Illumina Miniseq instruments will be carried out at UQCCR. Bacterial isolate identity (genus, species, sequence-type) and antimicrobial resistance genotype will be determined using the Queensland Genomics Infectious Disease (QGID) clinical genomics pipeline (https://github.com/FordeGenomics/SnapperRocks). In brief, in silico taxonomic profiling of isolates will be performed by screening the sequence read data against a bacterial refseq database using Kraken2. Sequence Types will be assigned using mlst and species-specific typing schemes available on PubMLST. Acquired antibiotic resistance genes will be identified by screening the draft genomes of each isolate against the NCBI resistance gene database using abricate. Finally, intrinsic mutation associated with antibiotic resistance will be identified using Pointfinder. For both technologies, time to pathogen identification, and time to susceptibility prediction will be measured. High quality complete reference genomes will be assembled from Illumina and MinION data using the Queensland Genomics Infectious Disease (QGID) reference genome assembly pipeline, Micropipe (www.biorxiv.org/content/biorxiv/early/2021/02/03/2021.02.02.429319.full.pdf). At least one complete genome will be assembled for each species identified clinically. The complete genomes will provide a gold-standard reference to evaluate any differences between direct clinical sample sequencing and the composite reference standard.

### ONT MinION Nanopore Sequencing

For the direct sequencing protocol, nucleic acid extracts will undergo short multiple-displacement amplification reactions followed by purification and concentration using Monarch PCR and DNA cleanup columns. The nucleic acid extract will be checked for DNA yield using a QBIT 3.0 instrument, followed by a nanopore library preparation using the ONT Rapid Sequencing Kit. The prepared sample will be loaded onto a MinION device using the R9.4.1 flow cell and run for 24 hours. Time-stamped fast5 files will be stored on UQ research data manager (RDM) for emulated real-time analysis. Taeper (https://github.com/mbhall88/taeper) will be used for emulating real-time sequencing for subsequent online analysis. The following computing steps will be carried out as a streaming pipeline based on the real-time data emulation: base-calling will be carried out using Guppy v4.0.11 run on high performance GPU (graphics processing unit) computing facilities at UQ; subsequent quality control with nanoq (https://github.com/esteinig/nanoq); species classification and identification of acquired resistance genes using JAPSA (https://github.com/mdcao/npAnalysis); and strain identification using Sketchy (https://github.com/esteinig/sketchy), for Klebsiella pneumoniae and Staphylococcus aureus identified infections. JAPSA calculates confidence intervals for the proportion of reads generated by each species identified. A species will be classified as identified if the 95% lower confidence interval of the proportion of reads from this species is greater than 0.

### Statistical Methods

The primary outcome measure, time to optimal antimicrobial therapy will be reported by median and interquartile range. Comparison between projected time to optimal antimicrobial therapy using an integrated diagnostic sequencing and dosing algorithm will be compared with the observed time to optimal therapy using a suitable non-parametric test such as the Kruskal-Wallis test. Diagnostic accuracy of MinION nanopore pathogen sequencing will be reported as sensitivity, specificity, positive and negative likelihood ratios against the composite reference standard reported above and illustrated by a 2x2 table. Appropriate methods to handle missing data will be developed following an investigation of the pattern of missingness. Where appropriate, multiple imputation will be used to replace missing values from a distribution of plausible values.

The full protocol, patient-level dataset and statistical code will be made available on reasonable request.

### Oversight and Monitoring

The study will be overseen by the Trial Steering Committee (TSC) comprised of experienced chief investigators from the participating ICUs (Kruger, Lipman, Schlapbach, Shekar), along with expertise in Infectious Diseases (Irwin), Microbiology (Bialasiewicz, Harris), Bioinformatics (Coin) and Pharmacy (Roberts). The TSC developed the protocol and meet monthly during the course of the trial. The TSC will evaluate any reports of adverse events (AEs), though few or no significant adverse events are anticipated in this study and a Data Safety and Monitoring Board (DSMB) was not convened. It is recognised that the patient population in the ICU will experience a number of aberrations in laboratory values, signs and symptoms due to the severity of the underlying disease and the impact of standard treatments in the ICU. These will not necessarily constitute AEs unless they are considered to be related to study treatment/procedures (ie. blood sampling and software-guided dosing) or in the Principal Investigator’s clinical judgement are not recognised events consistent with the patient’s underlying disease and expected clinical course. Therefore, reporting of AEs in this study will be restricted to events that occur during the study period (i.e., during and up to 48 hours after ceasing software-guided dosing), and which are considered to be related to study-specific procedures. Similarly, only serious adverse events (SAEs) that are reasonably suspected by the site principal investigator to be related to study-specific procedures and blood sample collection will be reported. Reporting of SAE in this study will be restricted to events that occur during the study period (i.e., during and up to 48 hours after ceasing software-guided dosing). SAEs will be reported to the steering committee within 24 hours of study staff becoming aware of the event. The SAE reports will then be forwarded to the HREC in accordance with local requirements. Patient eligibility, consents, and sample processing will be audited every 6 months at each site. An annual progress report will be submitted to the approving HREC & local RGOs. This process will be undertaken independently of the study sponsor. All protocol amendments will be agreed by the chief investigators, and submitted for ethical and local governance approval. Important protocol amendments will be updated on the published trials registry.

### Dissemination Plans

Results will be published in a peer-reviewed journal and presented at relevant conferences. A report will also be submitted to Queensland Health. All data will be non-identifiable and subgroup analyses will be presented in such a way as not to enable identification of participants and/or individual sites. Diagnostic accuracy will be reported in line with the Standards for Reporting of Diagnostic Accuracy Studies (STARD) statement (Bossuyt et al., 2015).

### Trial Status

Recruitment to the observational Phase 1 of the study commenced on 17th March 2020. Recruitment to the interventional Phase 2 commenced in March 2021 and recruitment is anticipated to finish on 30th September 2021.

## Discussion

Sepsis is a leading cause of preventable harm, and delays to appropriate antimicrobial therapy increase the risk of adverse outcomes. In critically ill patients, reducing the time to optimal therapy may yield significant improvements in sepsis mortality and ICU length of stay. Our innovative approach of combining rapid pathogen sequencing with antimicrobial dosing software has the potential to overcome the existing limitations of conventional diagnostics. Despite growing interest, and encouraging progress in the development of metagenomic sequencing for pathogen detection (Gu et al., 2021), there are few examples of its use in bloodstream infections or sepsis. A number of case studies (Bialasiewicz et al., 2019), or small series (Grumaz et al., 2016; Grumaz et al., 2020) suggest the potential of the approach in this time-critical context. One other clinical trial is currently evaluating next generation sequencing in adults with sepsis (Brenner et al., 2018), but no published protocols which include children. Our pilot study will yield key feasibility data to inform further, urgently needed sepsis studies.

## Data Availability Statement

The raw data supporting the conclusions of this article will be made available by the authors, without undue reservation.

## Ethics Statement

The studies involving human participants were reviewed and approved by Children’s Health Queensland Hospital and Health Service Human Research Ethics Committee (HREC) [HREC/19/QCHQ/55177]. Written informed consent to participate in this study was provided by the participants’ legal guardian/next of kin.

## Author Contributions

AI, LS, LC, and JR devised the study and led the development of the study protocol. MC and JR designed the methods to implement the dosing software intervention. LC, SB, and RB led the development of nanopore sequencing methods. SB led the development of QGID genome assembly pipelines. MB and PH led the development of methods for sequencing from culture broth. All authors contributed to the article and approved the submitted version.

## Funding

The DIRECT study is funded by the Queensland Genomics Health Alliance (QGHA) Round 2 (now Queensland Genomics) clinical implementation, innovation and incubation program and by a Brisbane Diamantina Health Partners Health System Improvement Ideas Grant (MRFF Rapid Applied Research Translation Program). ADI is supported by a NHMRC Emerging Leadership Investigator Award (APP1197743). LJS is supported by a NHMRC Practitioner Fellowship (APP1161657) and by the Children`s Hospital Foundation, Brisbane, Australia. PNAH is supported by a NHMRC Early Career Fellowship (APP1157530). JAR is supported by a NHMRC Practitioner Fellowship (APP1117065).

## Conflict of Interest

AI has received research funding and teaching honoraria from Gilead Sciences inc. unrelated to this work. DP has received research funding from Pfizer, Merck and Shionogi and funding for advisory boards or speaking engagements from Merck, Pfizer, BioMerieux, Sumitomo, Accelerate, QPex and Entasis, unrelated to this work. LC has received research funding from Oxford Nanopore Technologies unrelated to this work, and received travel reimbursement to travel to a conference. JR has consulted for or received grants from The Medicines Company, MSD, Biomerieux, QPEX, Pfizer and Discuva.

The remaining authors declare that the research was conducted in the absence of any commercial or financial relationships that could be construed as a potential conflict of interest.

## Abbreviations

AMR, antimicrobial resistance; AEs, adverse events; BMD, broth microdilution method; ID-ODS™, Bayesian dosing software (ID-ODS™); DNA, Deoxyriboneucleic acid; DSMB, Data Safety Monitoring Board; DIRECT, Optimising treatment outcomes for children and adults through rapid genome sequencing of sepsis pathogens; EDTA, Ethylenediamine Tetraacetic acid; EUCAST, European Committee on Antimicrobial Susceptibility Testing; GPU, Graphics processing unit; HREC, Human Research Ethics Committee; ICU, Intensive Care Unit; MRFF, Medical Research Future Fund; MinION, portable, real-time device for DNA and RNA sequencing; NATA, National Association of Testing Authorities; ONT, Oxford Nanopore Technologies; QGHA, Queensland Genomics Health Alliance; QGID, Queensland Genomics Infectious Disease; QPID, Queensland Paediatric Infectious Disease laboratory; PCR, Polymerase chain reaction; RDM, Research Data Manager; RGOs, Research Governance Officers; SAE, Serious Adverse Event; STARD, Standards for Reporting of Diagnostic Accuracy Studies; TSC, Trial Steering Committee; UQ, The University of Queensland; UQCCR, The University of Queensland Centre for Clinical Research.
