# Supplementary material for: Optimising Treatment Outcomes for Children and Adults Through Rapid Genome Sequencing of Sepsis Pathogens. A Study Protocol for a Prospective, Multi-Centre Trial (DIRECT)
Source: Front Cell Infect Microbiol. 2021 Jun 23;11:667680. doi: 10.3389/fcimb.2021.667680 (PMC8261237; doi:10.3389/fcimb.2021.667680)
Supplement: Supplementary file 1 [file DataSheet_1.docx]

**Title**

**Optimising treatment outcomes for children and adults through rapid genome sequencing of sepsis pathogens. A study protocol for a prospective, multi-centre trial (DIRECT)**

**Administrative information**

Note: the numbers in curly brackets in this protocol refer to SPIRIT checklist item numbers.^1^ The order of the items has been modified to group similar items (see <http://www.equator-network.org/reporting-guidelines/spirit-2013-statement-defining-standard-protocol-items-for-clinical-trials/>).

| Title {1} | **Optimising treatment outcomes for children and adults through rapid genome sequencing of sepsis pathogens. A study protocol for a prospective, multi-centre trial (DIRECT)** |
| --- | --- |
| Scientific Title | **Reducing time to appropriate antibiotics by integrating rapid pathogen genome sequencing with personalised antimicrobial dosing in children and adults with sepsis. A study protocol for a prospective, multi-centre trial (DIRECT)** |
| Trial registration {2a and 2b}. | Phase 2 of the trial protocol was registered with the Australia New Zealand Clinical Trials Registry on 30^th^ October 2020. ACTRN12620001122943 |
| Protocol version {3} | Version 5.0, 8^th^ November 2020 |
| Funding {4} | The DIRECT study is funded by the Queensland Genomics Health Alliance Round 2 clinical implementation, innovation and incubation program and by two Brisbane Diamantina Health Partners Health System Improvement Ideas Grants (Medical Research Future Fund (MRFF) Rapid Applied Research Translation Program). |
| Author details {5a} | Adam D. Irwin  Lachlan J.M Coin  Patrick N.A Harris  Menino Osbert Cotta  Michelle J Bauer  Cameron Buckley  Ross Balch  Peter Kruger  Jason Meyer  Kiran Shekar  Kara Brady  Cheryl Fourie  Natalie Sharp  Luminita Vlad  David Whiley  Scott A. Beatson  Brian M. Forde  David Paterson  Julia Clark  Krispin Hajkowicz  Sainath Raman  Seweryn Bialasiewicz  Jeffrey Lipman  Luregn J. Schlapbach*  Jason A. Roberts*  *Contributed equally |
| Name and contact information for the trial sponsor {5b} | The University of Queensland ABN 63 942 912 684 a body corporate constituted under the University of Queensland Act 1998 (Qld) of Brisbane in the State of Queensland 4072, Australia.  Joe McLean, Director, Research Partnerships  Level 2, Cumbrae-Stewart (Building 72) The University of Queensland Brisbane, QLD 4072  Email: director.partnerships@research.uq.edu.au |
| Role of sponsor {5c} | The study sponsor provides oversight and monitoring of the study. The study steering committee oversees the integrity and conduct of the study including reporting to the funders. Funders have no role in the design, conduct, data collection, management, analysis, and interpretation of data; writing of the report; or decision to submit the report for publication.  No significant adverse events are anticipated in this study and a Data Safety Monitoring Board (DSMB) was not required. |

**Contribution of the manuscript to the research field:**

Sepsis contributes significantly to morbidity and mortality globally, and prompt effective antimicrobial therapy is demonstrably important in improving outcomes. This protocol describes a multi-centre pilot study of an integrated diagnostic and therapeutic algorithm, in critically ill patients with sepsis. We take a highly novel approach to both diagnostic testing, using nanopore pathogen sequencing direct from clinical samples, and to achieving therapeutic drug levels with Bayesian dosing software. The trial will demonstrate the feasibility and diagnostic accuracy of pathogen sequencing direct from clinical samples, and estimate the impact of this approach on time to effective therapy when integrated with personalised software-guided antimicrobial dosing. in children and adults on ICU with sepsis. Clinical trials in sepsis are a substantial logistical challenge, and our multi-centre study of critically ill children and adults will additionally yield insights into the practicalities of performing a future definitive trial of the diagnostic and therapeutic algorithm.

**Introduction**

**Background and rationale {6a}**

Sepsis contributes significantly to morbidity and mortality of children and adults worldwide with an incidence that is increasing in line with global estimates.^2–4^ Close to 20,000 Australians develop sepsis every year, in whom approximately 5,000 deaths occur. The cost to the Australian healthcare system has been estimated to exceed AUD$846 million annually, with ICU treatment required for ongoing organ dysfunction resulting in the majority of costs (<https://www.georgeinstitute.org/sites/default/files/documents/stopping-sepsis-national-action-plan.pdf>). Appropriate antibiotic therapy is demonstrably effective in improving outcomes in sepsis. ^5–9^ However, the appropriateness of antibiotic therapy in sepsis is still predominantly determined by traditional susceptibility testing of cultured organisms. These conventional techniques have a yield below 50% in most ICU studies and take days to complete.^10^ It is often 48 to 72 hours before antibiotic susceptibility of pathogens are known, leading to substantial delays in optimizing antibiotic therapy, and to overuse of broad spectrum antibiotics. This timeframe for the identification of a causative organism and its susceptibility profile may result in a prolonged period of inappropriate empirical treatment. In an era of increasing antibiotic resistance, such suboptimal therapy can contribute to increased resistance, and worse patient-centred outcomes. In adults, it has been observed that Gram-negative healthcare-associated infections are associated with significant delays in effective antibiotic therapy. Such delays prolong the requirement for intensive care and lead to increased morbidity, mortality and cost.^11^ A day in ICU costs approximatively AUD$6,000 for ICU infrastructure alone. Hence strategies with the potential to hasten patient recovery and shorten ICU length of stay have great promise to be cost-effective.

A rapid, sensitive technique capable of identifying the pathogen responsible for sepsis, along with its susceptibility profile has the potential to substantially improve clinical outcomes through earlier selection of effective antimicrobial therapy. Culture-independent methods which are not limited to specific pathogen targets are appealing. Sequencing-based approaches meet these requirements. In particular, the Oxford Nanopore Technologies (ONT) MinION device provides informative sequence data in real-time,^12^ offering the potential for rapid pathogen identification along with susceptibility profiles. This effectiveness can be maximised by optimising antibiotic concentrations through individualized dosing.^13,14^

We propose an integrated approach to personalize antimicrobial therapy in children and adults with sepsis. We will evaluate the impact of portable nanopore sequencing using the ONT MinION device integrated with Bayesian dosing software (ID-ODS) to ensure achievement of maximally effective personalised antibiotic exposures in critically ill children and adults with sepsis.

**Objectives {7}**

**Hypothesis:**

Real-time pathogen sequencing combined with dosing software to identify optimised personalised antimicrobial therapy will reduce the time to effective antimicrobial concentrations in critically ill patients with sepsis leading to improved patient outcomes.

**Aim:**

To demonstrate the feasibility and diagnostic accuracy of real-time pathogen sequencing direct from clinical samples and estimate the impact of this diagnostic approach on time to effective therapy when integrated with personalised antimicrobial dosing in children and adults on ICU with sepsis.

**Objectives:**

**Primary objective:**

To estimate the reduction in time to effective antibiotic therapy in children and adults with sepsis using rapid pathogen sequencing combined with dosing software when compared to conventional diagnostic and therapeutic regimens.

**Secondary objectives:**

1. A demonstration of the feasibility of real-time pathogen sequencing in whole blood samples from critically ill children and adults with sepsis.
2. An evaluation of the diagnostic accuracy of real-time pathogen sequencing from positive blood cultures taken from critically ill children and adults with sepsis.
3. An evaluation of the time to pathogen identification comparing direct sequencing versus conventional blood cultures taken from critically ill children and adults with sepsis.
4. An improvement in the achievement of therapeutic antibiotic concentrations in patients using dosing software versus traditional dosing regimens.

The primary outcome is time to effective antimicrobial therapy, defined as trough drug concentrations above the MIC of the pathogen. Secondary outcomes are diagnostic accuracy of direct pathogen sequencing from whole blood, and time to pathogen identification and susceptibility testing using pathogen sequencing direct from whole blood and from positive blood culture broth.

**Trial design {8}**

The DIRECT study is a pilot prospective, non-randomised multicentre trial of an integrated diagnostic and therapeutic algorithm combining rapid direct pathogen sequencing and software-guided, personalised antibiotic dosing in children and adults with sepsis on ICU.

It includes a diagnostic accuracy study of real-time pathogen sequencing direct from patient blood using the ONT MinION device, which will be compared to conventional pathogen sequencing of cultured isolates, and conventional blood culture and phenotypic susceptibility testing.

**Methods: Participants, interventions and outcomes**

**Study setting {9}**

The study will be undertaken in the following participating ICUs in Brisbane, Australia:

Royal Brisbane and Women’s Hospital
The Prince Charles Hospital
Princess Alexandra Hospital
Queensland Children’s Hospital

**Eligibility criteria {10}**

**Inclusion criteria:**

1. Age >1month
2. Admitted to paediatric or adult ICU at one of the participating centres
3. Decision to treat for suspected sepsis, defined as suspected or proven infection or without confirmed organ dysfunction.
4. Commenced within 24h on intravenous broad-spectrum antibiotics, or within 24h of a change to new antibiotics consistent with treatment for a new episode of suspected sepsis.
5. Blood cultures are being obtained or were obtained within the past 12 hours

**Exclusion criteria:**

1. Inability to gain informed consent during the study period
2. Neonates
3. Death is likely imminent
4. Palliative care patient
5. Renal replacement therapy
6. Extra-corporeal membrane oxygenation

**Who will take informed consent? {26a}**

Patients and/or carers will be approached by clinical research staff at each participating ICU and provided with written information regarding the study. A consent to continue process will be used where prospective recruitment would lead to delays in blood sampling and preclude direct comparison with blood cultures. Carers and competent patients will be provided with the opportunity to revoke their consent and have the research blood samples and data securely disposed of prior to analysis.

**Additional consent provisions for collection and use of participant data and biological specimens {26b}**

Eligible patients and their carers will be approached to contribute residual blood samples for the evaluation of novel, rapid sepsis diagnostics (commercial and research, culture-dependent and culture-independent).

**Interventions**

**Explanation for the choice of comparators {6b}**

Diagnostic accuracy of MinION nanopore pathogen sequencing will be compared with conventional blood cultures and antimicrobial susceptibility testing. Acknowledging the limited yield of blood cultures in sepsis, a composite reference standard for sepsis will be used. This composite reference standard will incorporate blood culture results, other significant microbiological samples taken at the discretion of the clinical team and clinical and epidemiological features. These composite features will be interpreted independently by two experts in microbiology and infectious disease. In the event of discordance a third expert opinion will adjudicate.

Complete reference genomes for matching pathogen isolates will be analysed to evaluate the differences between nanopore direct sequencing and cultured pathogens sequenced conventionally.

**Intervention description {11a}**
Phase 1:

In an initial observational phase of the study, participants will be recruited to a diagnostic accuracy study of MinION nanopore pathogen sequencing. The sample size for this phase of the study is 50 patients with blood culture-confirmed sepsis admitted to ICU.

Phase 2:

In Phase 2, consecutive patients with suspected sepsis admitted to ICU will undergo MinION nanopore pathogen sequencing integrated with personalised antibiotic therapy using a combination of Bayesian dosing software (ID-ODS^TM^) and measured antibiotic plasma concentrations. A senior ICU pharmacist/clinician at each site will lead this software-guided intervention of antimicrobial dose optimisation. All dosing regimens will be checked by both the senior ICU pharmacist and attending ICU consultant prior to prescription. The final decision regarding the use of the optimised dosing of antibiotics will remain at the discretion of the attending ICU consultant.

**Figure 1: Study flow diagram**

**Criteria for discontinuing or modifying allocated interventions {11b}**

Software-guided dosing will continue until either: 1) the study antibiotic(s) have been ceased by the treating clinician, 2) the patient is discharged from ICU, 3) after 5 days of study antibiotic therapy. If antibiotic therapy is still required thereafter, dosing will be guided by the treating clinician.

**Strategies to improve adherence to interventions {11c}**

Adherence to dosing strategies informed by the dosing software will be supported by the use of senior ICU pharmacists trained in the use of this software-guided approach to antimicrobial dose optimisation. All dosing regimens will be checked by both the senior ICU pharmacist and attending ICU consultant prior to prescription, to ensure appropriateness and safety.

**Relevant concomitant care permitted or prohibited during the trial {11d}**

Not applicable

**Provisions for post-trial care {30}**

Not applicable

**Outcomes {12}**

**Primary outcome:**

The primary outcome is time to effective antimicrobial therapy defined as trough drug concentrations above the MIC of the pathogen.

**Secondary outcomes:**

Secondary outcomes are:

1. Diagnostic accuracy of MinION nanopore pathogen sequencing direct from whole blood
2. Time to pathogen identification and susceptibility testing using MinION nanopore pathogen sequencing
   1. direct from whole blood
   2. from positive blood culture broth.
3. Evaluation of direct MinION nanopore pathogen sequencing predictive capabilities using complete reference genomes from positive blood culture broth.

**Participant timeline {13}**

Recruitment to the study commenced 17th March 2020 and will be completed by 1^st^ April 2021.

**Sample size {14}**

The study will be undertaken in two phases. Phase 1 will seek to demonstrate the validity of the sequencing method applied to patients with blood culture-confirmed sepsis (n=50). Recruitment of 50 blood culture confirmed-sepsis patients in this phase is anticipated to require the recruitment of approximately 150 patients with suspected sepsis. Phase 2 will then apply these methods to consecutive patients (n=50) admitted to intensive care with suspected sepsis.

**Recruitment {15}**

The study Steering Committee includes chief investigators from each participating ICU. Education sessions were conducted with research groups from each ICU in advance of the study onset, including individual sessions with local research coordinators. Study sample packs, and flow diagrams to aid recruitment were created for participating ICUs. Research coordinators are responsible for screening potentially eligible patients with suspected sepsis on a daily basis. Finally, ethical approval was sought to recruit patients with suspected sepsis on a “consent to continue” basis, reflecting the time-critical nature of sepsis and the importance of obtaining timely research samples.

**Assignment of interventions: allocation**

No randomization of patients will occur.

**Sequence generation {16a}**

Not applicable

**Concealment mechanism {16b}**

Not applicable

**Implementation {16c}**

Not applicable

**Assignment of interventions: Blinding**

**Who will be blinded {17a}**

Not applicable

**Procedure for unblinding if needed {17b}**

Not applicable

**Data collection and management**

**Plans for assessment and collection of outcomes {18a}**

Clinical data on patient demographics, comorbidities, type and focus of infection, severity, clinical progress through ICU and clinical outcomes (including ICU and hospital length of stay and in-hospital mortality) will be captured by clinical research nurses into an online REDCap database hosted by The University of Queensland. Organ dysfunction in children and adults is defined in Appendix 1.

Initial study samples will be obtained during the course of the routine clinical sepsis workup. Samples will be stored until consent to participate has been sought, and will then be analysed if consent is given, or destroyed if consent is not given or revoked.

The following study samples will be obtained:

1. A single study sample will be taken into an EDTA container at the time of recruitment to the study, and as close to the time of initial blood culture sampling on suspicion of sepsis. (Time=zero). Routine clinical sampling should include appropriate volumes of blood and other clinical samples for culture and susceptibility testing.
2. Plasma samples will be taken at the following time points of antibiotic therapy: at 24 hours, 48 hours, 72 hours and 96 hours post-commencement of antibiotic treatment to determine if target antibiotic plasma concentrations have been reached.

Clinical samples will be processed in line with existing laboratory requirements. Study EDTA samples will be stored at 4°C before shipping to The University of Queensland Centre for Clinical Research (UQCCR) and the Queensland Paediatric Infectious Disease (QPID) laboratory. Bacterial isolates will be stored at -80°C before shipping to UQCCR. Samples will be de-identified before shipping, and labelled with a study ID. Samples at UQCCR will be stored at -80°C until analysis.

Appropriate study sample volumes depending on age:

Initial EDTA sample (T=0)

Infants and Young children (<5y): 1-2ml
Older children (5-12y): 2-6ml
Adolescents and adults: 6-10ml

Subsequent plasma samples for antimicrobial dosing (T=24, 48, 72, 96h)

Infants and Young children (<5y): 0.5ml
Older children (5-12y): 1-2ml
Adolescents and adults: 3-5ml

The volume of blood obtained (for both children and adults) will be documented at the time of recruitment and sampling. Consent will be sought to use residual EDTA samples for the evaluation of emerging, rapid methods of bacterial detection and antimicrobial resistance identification.

**Reference standard**

The diagnostic accuracy of real-time pathogen sequencing using the MinION sequencer for the diagnosis of sepsis will be evaluated against a reference standard of conventional microbiological evaluation using blood culture and susceptibility testing. Routine clinical samples will be processed in NATA accredited laboratories at Pathology Queensland. Time to blood culture growth, time to pathogen identification, and time to susceptibility testing completion will be collected.

**Broth microdilution**

Susceptibility testing will be undertaken at UQCCR using a broth microdilution method (BMD). In house BMD plates will be made using the Hamilton Star liquid handling robot as per European Committee on Antimicrobial Susceptibility Testing (EUCAST) standards and testing will be performed on demand as isolates are made available. Antimicrobials tested by BMD will be chosen by the study investigators and will reflect available and readily used antimicrobials in clinical practice.

**Whole genome sequencing of sepsis pathogens**

Whole genome sequencing (WGS) of sepsis pathogen isolates with ONT MinION or Illumina Miniseq instruments will be carried out at UQCCR. Bacterial isolate identity (genus, species, sequence-type) and antimicrobial resistance genotype will be determined using the Queensland Genomics Infectious Disease (QGID) WGS pipeline. For both technologies, time to pathogen identification, and time to susceptibility prediction will be measured. High quality complete reference genomes will be assembled from Illumina and MinION data using the Queensland Genomics Infectious Disease (QGID) reference genome assembly pipeline. The complete genomes will provide a gold-standard reference to evaluate any differences between direct clinical sample sequencing and the composite reference standard.

**Pathogen sequencing direct from clinical samples**

**Human DNA depletion and nucleic acid extraction**

Bacterial cells will be enriched using a differential lysis procedure. Residual human DNA will be degraded using a DNAse treatment, followed by nucleic acid extraction using a rapid commercial-kit based protocol.

**ONT MinION nanopore sequencing**

For the direct sequencing protocol, nucleic acid extracts will undergo short multiple-displacement amplification reactions followed by purification and concentration using Monarch PCR and DNA cleanup columns. The nucleic acid extract will be checked for DNA yield using a QBIT 3.0 instrument, followed by a nanopore library preparation using the ONT Rapid Sequencing Kit. The prepared sample will be loaded onto a MinION device using the R9.4.1 flow cell and run for 24 hours. Time-stamped fast5 files will be stored on UQ research data manager (RDM) for emulated real-time analysis. Taeper ( <https://github.com/mbhall88/taeper>) will be used for emulating real-time sequencing for subsequent online analysis. The following computing steps will be carried out as a streaming pipeline based on the real-time data emulation: base-calling will be carried out using Guppy v4.0.11 run on high performance GPU (graphics processing unit) computing facilities at UQ; subsequent quality control with nanoq (<https://github.com/esteinig/nanoq>); species classification and identification of acquired resistance genes using JAPSA (<https://github.com/mdcao/npAnalysis>); and strain identification using Sketchy (<https://github.com/esteinig/sketchy> ), for Klebsiella pneumoniae and Staphylococcus aureus identified infections. JAPSA calculates confidence intervals for the proportion of reads generated by each species identified. A species will be classified as identified if the 95% lower confidence interval of the proportion of reads from this species is greater than 0.

**Rapid pathogen detection and antimicrobial resistance identification**

**Residual samples not required for use in the MinION nanopore sequencing study will be salvaged for the evaluation of other emerging rapid detection technologies that may become available during the course of the study, such as T2 biosystems. This will not require any further sample collection from patients enrolled in the study.**

**Plans to promote participant retention and complete follow-up {18b}**

Not applicable

**Data management {19}**

Data will be entered directly into an online REDCap database hosted by the University of Queensland. In the event that a paper Case report form is used, forms will be stored in an appropriate locked storage facility with restricted access. A study ID number will be used to link de-identified clinical data with the laboratory samples. Deidentified genome sequence data will be shared by deposition in a public nucleotide repository (GenBank and Sequence Read Archive).

Data and samples will be retained in line with the Queensland Health Retention and Disposal Schedule and with the Australian Code for the Responsible Conduct of Research Section 2.1.1.

**Data monitoring**

Adherence to the study protocol will be monitored by the Coordinating Principal Investigator, and the steering committee. Patient eligibility, consents, and sample processing will be audited every 6 months at each site. An annual progress report will be submitted to the approving human research ethics committee (HREC) and local research governance officers (RGOs). No adverse events are anticipated, and a Data Safety an\d Monitoring Board has not been convened.

**Confidentiality {27}**

Clinical data on patient demographics, comorbidities, type and focus of infection, severity, clinical progress through ICU and clinical outcomes (including ICU and hospital length of stay and in-hospital mortality) will be captured by clinical research nurses into a secure, password-restricted online REDCap database hosted by The University of Queensland. A study ID number will be used to link de-identified clinical data with the laboratory samples.

**Plans for collection, laboratory evaluation and storage of biological specimens for genetic or molecular analysis in this trial/future use {33}**

**Statistical methods
Statistical methods for primary and secondary outcomes {20a}**

The primary outcome measure, time to optimal antimicrobial therapy will be reported by median and interquartile range. Comparison between projected time to optimal antimicrobial therapy using an integrated diagnostic sequencing and dosing algorithm will be compared with the observed time to optimal therapy using a suitable non-parametric test such as the Kruskal-Wallis test.

Diagnostic accuracy of MinION nanopore pathogen sequencing will be reported as sensitivity, specificity, positive and negative likelihood ratios against the composite reference standard reported above and illustrated by a 2x2 table.

**Interim analyses {21b}**

Not applicable

**Methods for additional analyses (e.g. subgroup analyses) {20b}**

Not applicable

**Methods in analysis to handle protocol non-adherence and any statistical methods to handle missing data {20c}**

Appropriate methods to handle missing data will be developed following an investigation of the pattern of missingness. Where appropriate, multiple imputation will be used to replace missing values from a distribution of plausible values

**Plans to give access to the full protocol, participant level-data and statistical code {31c}**

The full protocol, patient-level dataset and statistical code will be made available on reasonable request.

**Oversight and monitoring**

**Composition of the coordinating centre and Trial Steering Committee {5d}**

Luregn Schlapbach Intensive Care

Adam Irwin Infectious Diseases

Lachlan Coin Bioinformatics

Patrick Harris Microbiology

Seweryn Bialasiewicz Microbiology

Kiran Shekar Intensive Care

Peter Kruger Intensive Care

Jeffrey Lipman Intensive Care

Jason Roberts Pharmacy

The Trial Steering Committee (TSC) is comprised of experienced chief investigators from the participating ICUs, along with expertise in Infectious Diseases, Microbiology, and Pharmacy. The TSC developed the protocol and meet monthly during the course of the trial. The TSC will evaluate any reports of adverse events.

**Composition of the data monitoring committee, its role and reporting structure {21a}**

No significant adverse events are anticipated in this study and a Data Safety Monitoring Board (DSMB) was not convened.

**Adverse event reporting and harms {22}**

Adverse events (AEs) are defined as any untoward medical occurrence in a participant administered the study intervention which does not necessarily have to have a causal relationship with the study treatment. Few or no adverse events are anticipated, and a DSMB is not required.

It is recognised that the patient population in the ICU will experience a number of aberrations in laboratory values, signs and symptoms due to the severity of the underlying disease and the impact of standard treatments in the ICU. These will not necessarily constitute AEs unless they are considered to be related to study treatment/procedures (ie. blood sampling and software-guided dosing) or in the Principal Investigator’s clinical judgement are not recognised events consistent with the patient’s underlying disease and expected clinical course. Therefore, reporting of AEs in this study will be restricted to events that occur during the study period (i.e., during and up to 48 hours after ceasing software-guided dosing), and which are considered to be related to study-specific procedures.

**Serious adverse events (SAE)**

Serious adverse events (SAEs) are defined as any untoward medical occurrence that meets one or more of the following criteria:

- Is life-threatening, or results in persistent or significant disability or death;
- Requires prolongation of existing hospitalisation;
- Requires intervention to prevent one of the previously listed outcomes.

The classification of SAE is not related to the assessment of the severity of the adverse event. An event that is mild in severity may be classified as a serious adverse event based on the above criteria. Given that critically ill patients are likely to experience any of the above listed criteria in the course of their ICU admission, only serious adverse events that are reasonably suspected by the site principal investigator to be related to study-specific procedures and blood sample collection will be reported. Reporting of SAE in this study will be restricted to events that occur during the study period (i.e., during and up to 48 hours after ceasing software-guided dosing).

SAEs will be reported to the steering committee within 24 hours of study staff becoming aware of the event. The SAE reports will then be forwarded to the HREC in accordance with local requirements.

**Frequency and plans for auditing trial conduct {23}**

Patient eligibility, consents, and sample processing will be audited every 6 months at each site. An annual progress report will be submitted to the approving HREC & local RGOs. This process will be undertaken independently of the study sponsor.

**Plans for communicating important protocol amendments to relevant parties (e.g. trial participants, ethical committees) {25}**

All protocol amendments will be agreed by the chief investigators, and submitted for ethical and local governance approval. Important protocol amendments will be updated on the published trials registry.

**Dissemination plans {31a}**

Results will be published in a peer-reviewed journal and presented at relevant conferences. A report will also be submitted to Queensland Health. All data will be non-identifiable and subgroup analyses will be presented in such a way as not to enable identification of participants and/or individual sites. Diagnostic accuracy will be reported in line with the Standards for Reporting of Diagnostic Accuracy Studies (STARD) statement.^15^

**Trial status**

Recruitment to the observational Phase 1 of the study commenced on 17^th^ March 2020. Recruitment to the interventional Phase 2 has not yet commenced and recruitment is anticipated to finish on 1^st^ June 2021.

**Abbreviations**

**AMR:** antimicrobial resistance

**AEs:** adverse events

**BMD:** broth microdilution method

**ID-ODS^TM^:** Bayesian dosing software (ID-ODS^TM^)

**DNA**: Deoxyriboneucleic acid

**DSMB:** Data Safety Monitoring Board

**DIRECT:** Optimising treatment outcomes for children and adults through rapid genome sequencing of sepsis pathogens

**EDTA:** Ethylenediamine Tetraacetic acid.

**EUCAST:** European Committee on Antimicrobial Susceptibility Testing

**GPU:** Graphics processing unit

**HREC:** Human Research Ethics Committee

**ICU:** Intensive Care Unit

**MRFF:** Medical Research Future Fund

**MinION:** portable, real-time device for DNA and RNA sequencing

**NATA:** National Association of Testing Authorities

**ONT:** Oxford Nanopore Technologies

**QGHA:** Queensland Genomics Health Alliance

**QGID:** Queensland Genomics Infectious Disease

**QPID:** Queensland Paediatric Infectious Disease laboratory

**PCR:** Polymerase chain reaction

**RDM:** Research Data Manager

**RGOs:** Research Governance Officers

**SAE:** Serious Adverse Event

**STARD:** Standards for Reporting of Diagnostic Accuracy Studies

**TSC:** Trial Steering Committee

**UQ:** The University of Queensland

**UQCCR:** The University of Queensland Centre for Clinical Research

**Declarations**

**Acknowledgements**

**Authors’ contributions {31b}**

ADI, LJS, LC and JAR devised the study and led the development of the study protocol.

MOC and JAR designed the methods to implement the dosing software intervention

LC, SB and RB led the development of nanopore sequencing methods.

SAB led the development of QGID genome assembly pipelines.

M L-B and PNAH led the development of methods for sequencing from culture broth.

All named investigators contributed to and approved the submitted protocol.

**Funding {4}**

The DIRECT study is funded by the Queensland Genomics Health Alliance (QGHA) Round 2 (now Queensland Genomics) clinical implementation, innovation and incubation program and by a Brisbane Diamantina Health Partners Health System Improvement Ideas Grant (MRFF Rapid Applied Research Translation Program).

ADI is supported by a NHMRC Emerging Leadership Investigator Award.

LJS is supported by a practitioner fellowship by NHMRC and by the Children`s Hospital Foundation, Brisbane, Australia.

PNAH is supported by a NHMRC Early Career Fellowship.

JAR is supported by a NHMRC Practitioner Fellowship.

**Availability of data and materials {29}**

The full dataset will be available to all chief investigators. Access to the full dataset will be made available on reasonable request.

**Ethics approval and consent to participate {24}**

Ethics approval has been granted by the Children’s Health Queensland Hospital and Health Service Human Research Ethics Committee (HREC) [HREC/19/QCHQ/55177]. Written informed consent will be obtained from all participants (or their parent or legal guardian). Approval was granted by the Queensland Civil and Administrative Tribunal [CRL024-19] to include patients unable to consent for themselves under the Guardianship and Administration Act 2000.

**Consent for publication {32}**

**Competing interests {28}**

ADI has received research funding and teaching honoraria from Gilead Sciences inc. unrelated to this work.

DLP has received research funding from Pfizer, Merck and Shionogi and funding for advisory boards or speaking engagements from Merck, Pfizer, BioMerieux, Sumitomo, Accelerate, QPex and Entasis, unrelated to this work.

LC has received research funding from Oxford Nanopore Technologies unrelated to this work, and received travel reimbursement to travel to a conference.

JAR has consulted for or received grants from The Medicines Company, MSD, Biomerieux, QPEX, Pfizer and Discuva.

**Authors’ information (optional)**

**References**

1. Chan, A.-W. *et al.* SPIRIT 2013 Statement: Defining Standard Protocol Items for Clinical Trials. *Ann. Intern. Med.* **158**, 200 (2013).

2. Schlapbach, L. J. *et al.* Mortality related to invasive infections, sepsis, and septic shock in critically ill children in Australia and New Zealand, 2002–13: a multicentre retrospective cohort study. *Lancet Infect. Dis.* **15**, 46–54 (2015).

3. Fleischmann-Struzek, C. *et al.* The global burden of paediatric and neonatal sepsis: a systematic review. *Lancet Respir Med* **6**, 223–230 (2018).

4. Rudd, K. E. *et al.* Global, regional, and national sepsis incidence and mortality, 1990–2017: analysis for the Global Burden of Disease Study. *The Lancet* **395**, 200–211 (2020).

5. Rhodes, A. *et al.* Surviving Sepsis Campaign: International Guidelines for Management of Sepsis and Septic Shock. *Crit. Care Med.* **45**, 486–552 (2017).

6. Kumar, A. *et al.* Initiation of inappropriate antimicrobial therapy results in a fivefold reduction of survival in human septic shock. *Chest* **136**, 1237–48 (2009).

7. Seymour, C. W. *et al.* Time to Treatment and Mortality during Mandated Emergency Care for Sepsis. *N. Engl. J. Med.* **376**, 2235–2244 (2017).

8. Evans, I. V. R. *et al.* Association Between the New York Sepsis Care Mandate and In-Hospital Mortality for Pediatric Sepsis. *JAMA* **320**, 358 (2018).

9. Weiss, S. L. *et al.* Surviving sepsis campaign international guidelines for the management of septic shock and sepsis-associated organ dysfunction in children. *Intensive Care Med.* **46**, 10–67 (2020).

10. Agyeman, P. K. A. *et al.* Epidemiology of blood culture-proven bacterial sepsis in children in Switzerland: a population-based cohort study. *Lancet Child Adolesc Health* **1**, 124–133 (2017).

11. Moehring, R. W. *et al.* Delays in appropriate antibiotic therapy for gram-negative bloodstream infections: a multicenter, community hospital study. *PLoS One* **8**, e76225–e76225 (2013).

12. Clarke, J. *et al.* Continuous base identification for single-molecule nanopore DNA sequencing. *Nat. Nanotechnol.* **4**, 265–270 (2009).

13. Roberts, J. A. *et al.* Individualised antibiotic dosing for patients who are critically ill: challenges and potential solutions. *Lancet Infect. Dis.* **14**, 498–509 (2014).

14. the Infection Section of European Society of Intensive Care Medicine (ESICM) *et al.* Antimicrobial therapeutic drug monitoring in critically ill adult patients: a Position Paper#. *Intensive Care Med.* **46**, 1127–1153 (2020).

15. Bossuyt, P. M. *et al.* STARD 2015: an updated list of essential items for reporting diagnostic accuracy studies. *BMJ* **351**, h5527 (2015).

16. Singer, M. *et al.* The Third International Consensus Definitions for Sepsis and Septic Shock (Sepsis-3). *JAMA* **315**, 801 (2016).

17. Matics, T. J. & Sanchez-Pinto, L. N. Adaptation and Validation of a Pediatric Sequential Organ Failure Assessment Score and Evaluation of the Sepsis-3 Definitions in Critically Ill Children. *JAMA Pediatr.* **171**, e172352 (2017).
