# Supplementary material for: Optimising Treatment Outcomes for Children and Adults Through Rapid Genome Sequencing of Sepsis Pathogens. A Study Protocol for a Prospective, Multi-Centre Trial (DIRECT)
Source: Front Cell Infect Microbiol. 2021 Jun 23;11:667680. doi: 10.3389/fcimb.2021.667680 (PMC8261237; doi:10.3389/fcimb.2021.667680)
Supplement: Supplementary file 2 [file DataSheet_2.docx]

**Supplementary File 2: Definitions of organ dysfunction in children and adults**^16,17^**:**

1. For adults organ dysfunction as defined by having:

(a) at least 2 of the following:

- - - Pa0_2_ / FI0_2_ mm Hg ≤ 400
    - MAP <70mmHg in last 24 hours or received inotropes/vasopressors for at least 1 hour
    - Platelets <150
    - Creatinine >110
    - Bilirubin >20

OR

(b) a SOFA score of ≥2 not including SOFA CNS

1. For children organ dysfunction as defined by having:

(a) at least 2 of the following:

- - - Receiving invasive or non-invasive (not including HiFlow)
    - Platelets ≤ 150
    - Bilirubin ≥ 20umol/L
    - GCS <15
    - Hypotension with MAP below:
      - - 1-11mo: ≤55mmHg
        - 1-4y: ≤ 60mmHg
        - 12-18y: ≤ 67mmHg in last 24 hours
        - Or received inotropes / vasosopressors for at least 1 hour
    - Creatinine above:
- 1-11mo: ≥44umol/L
- 12-23mo: ≥53umol/L
- 2-4y: ≥80umol/L
- 5-12y: ≥ 97umol/L
- 12-18y: ≥150 umol/L

OR

(b) a pSOFA score of ≥2 not including pSOFA CNS
